# Effects of electrode size and placement on comfort and efficiency during low-intensity neuromuscular electrical stimulation of quadriceps, hamstrings and gluteal muscles

**DOI:** 10.1186/s13102-022-00403-7

**Published:** 2022-01-16

**Authors:** J. Flodin, R. Juthberg, P. W. Ackermann

**Affiliations:** 1 Section of Orthopedics and Sports Medicine, Integrative Orthopedic Laboratory, Department of Molecular Medicine and Surgery, Karolinska Institutet, Stockholm, Sweden; 2 Department of Trauma, Acute Surgery and Orthopaedics, Karolinska University Hospital, 171 76 Stockholm, Sweden

**Keywords:** Electric stimulation therapy, Pain threshold, Patient comfort, Electrode configuration, Skeletal muscles

## Abstract

**Background:**

Neuromuscular electrical stimulation (NMES) may prevent muscle atrophy, accelerate rehabilitation and enhance blood circulation. Yet, one major drawback is that patient compliance is impeded by the discomfort experienced. It is well-known that the size and placement of electrodes affect the comfort and effect during high-intensity NMES. However, during low-intensity NMES the effects of electrode size/placement are mostly unknown. Therefore, the purpose of this study was to investigate how electrode size and pragmatic placement affect comfort and effect of low-intensity NMES in the thigh and gluteal muscles.

**Methods:**

On 15 healthy participants, NMES-intensity (mA) was increased until visible muscle contraction, applied with three electrode sizes (2 × 2 cm, 5 × 5 cm, 5 × 9 cm), in three different configurations on quadriceps and hamstrings (short-transverse (ST), long-transverse (LT), longitudinal (L)) and two configurations on gluteus maximus (short-longitudinal (SL) and long-longitudinal (LL)). Current–density (mA/cm^2^) required for contraction was calculated for each electrode size. Comfort was assessed with a numerical rating scale (NRS, 0–10). Significance was set to *p* < 0.05 and values were expressed as median (inter-quartile range).

**Results:**

On quadriceps the LT-placement exhibited significantly better comfort and lower current intensity than the ST- and L-placements. On hamstrings the L-placement resulted in the best comfort together with the lowest intensity. On gluteus maximus the LL-placement demonstrated better comfort and required less intensity than SL-placement. On all muscles, the 5 × 5 cm and 5 × 9 cm electrodes were significantly more comfortable and required less current–density for contraction than the 2 × 2 cm electrode.

**Conclusion:**

During low-intensity NMES-treatment, an optimized electrode size and practical placement on each individual muscle of quadriceps, hamstrings and gluteals is crucial for comfort and intensity needed for muscle contraction.

**Supplementary Information:**

The online version contains supplementary material available at 10.1186/s13102-022-00403-7.

## Introduction

Neuromuscular electrical stimulation (NMES) is clinically used for rehabilitation, muscle strengthening [1, 2] and recovery after exercise [3, 4]. For muscle strengthening purposes, high-intensity (HI) NMES (≥ 45 mA) is required [2]. However, one major drawback to HI-NMES is that many patients experience discomfort resulting in low compliance [1, 2].

Low-intensity (LI) NMES (≤ 20 mA), at levels equal to a visible muscle contraction, improve the comfort compared to HI-NMES [2, 5]. LI-NMES applied on the lower limbs is known to increase blood circulation [6–8], enhance recovery after exercise [3, 4], and improve walking ability among older adults [5]. However, despite being more comfortable than HI-NMES, LI-NMES may still be perceived as uncomfortable by several patients. Therefore, it is important to investigate how other factors beyond intensity influence the comfort and effectiveness during LI-NMES.

When using HI-NMES, electrode size and placement clearly affect patient comfort and muscle force production [1, 2, 9–13]. Previous studies have indicated that larger electrodes during HI-NMES provide better comfort [9–12]. However, corresponding studies on electrode size have, to the best of our knowledge, not been performed for LI-NMES. Regarding the placement of electrodes, prior research evidently show that identification of and placement of electrodes on motor points improve patient comfort and enhance the muscle activating effect of NMES [2, 14, 15]. However, for patients using NMES at home, a motor point search can be quite complicated to perform, and many patients rather use predetermined placements.

Predetermined placements with HI-NMES suggest that a longer distance between the two electrodes, preferentially with longitudinal placement along muscle fibers at opposing ends [1], produces a greater muscle torque [13]. In contrast, another study using LI-NMES on the quadriceps muscle suggested that a relatively shorter longitudinal distance produced a greater knee extension force [16]. It is essential to find the best predetermined placements of electrodes during LI-NMES on the thigh and gluteal muscles to simplify NMES-usage for patients at home.

The hypothesis of this study was that LI-NMES can be administered with good comfort if optimized predetermined electrode placements and sizes are identified on major lower limb muscles. The purpose of this study was to investigate comfort and current intensity at the first visible muscle contraction during LI-NMES, among three different electrode sizes and three commonly used placements on quadriceps and hamstrings, and two placements on gluteus maximus.

## Methods

### Participants

A total of 15 healthy participants participated in the study (Table 1). Participants aged between 18 and 70 years were eligible for inclusion. All participants signed an informed consent, completed a questionnaire on the participant characteristics (age, sex, use of tobacco products and physical activity level) and the participants were measured in height and weight before the study. The exclusion criteria were pregnancy, skin ulcer, pacemaker, intracardiac defibrillator, advanced heart disease, kidney failure and neuromuscular or metabolic disease. None of the patients were smokers or used other tobacco products. The physical activity was estimated according to the Frändin/Grimby activity scale with a gradual increase of exercise from 1 to 6 [17]. The median score was 5, indicating exercise for three or more hours a week.

**Table 1:** Participant characteristics

Variable	Participant characteristics (n = 15)
Sex, female, n (%)	10 (66)
Age (years)	37 (24–51)^a^
Height (cm)	172 (169–180)^a^
Weight (kg)	68 (62–79.5)^a^
BMI (kg/cm^2^)	23 (21.2–24.5)^a^
Smoker, n (%)	0 (0)
Physical activity level (1–6)	5 (4–5)^a^

^a^Data are expressed as median (inter-quartile range)

### Assessment of comfort

The primary outcome variable was the level of comfort for each placement and size of the electrodes, which was measured using the Numeric rating scale for pain (NRS) with 0 indicating no discomfort or pain and 10 indicating the worst imaginable discomfort or pain [18]. NRS was rated by the participant for each increase in NMES-level and for each electrode placement until a visible muscle contraction occurred. The investigator recorded NRS on a prefabricated survey form, where the y-axis scale indicated the NRS, and the x-axis indicated the NMES-level.

### NMES-settings

A constant-current NMES-device, Chattanooga Physio (DJO Nordic, Malmoe, Sweden), was used for all the tests. The waveform of the current delivered was a symmetrical, biphasic, square-form pulse. The settings were the same for all tests, using a frequency of 36 Hz, phase duration of 400 µs (800 µs biphasic pulse), contraction time of 4 s (including 0.5 s ramp-up/-down time) and 8 s rest in between. The settings were based on previous findings [2, 19] as well as on our own pilot experiments.

#### Intensity of NMES

The NMES-level (0–999), representing a non-linear relationship to the stimulation intensity ranging from 0 to 120 milliampere (mA), was gradually increased by two NMES-levels at a time. The NMES-levels where the first visible muscle twitch and the first visible muscle contraction occurred were registered. The NMES-level needed for each test was translated into mA based on information from DJO Global. The current density can be calculated based on the intensity in mA and the area of the electrodes, intensity/area (mA/cm^2^).

### Electrode size

Self-adhesive electrodes (Compex Snap, Performance, DJO Global, USA) were used for the tests. For each test two electrodes of the same size were used.

Electrodes sized 2 × 2 cm, 5 × 5 cm and 5 × 9 cm were used. The electrodes sized 2 × 2 cm were not available to buy and was therefore crafted by trimming/cutting down the edges of 5 × 5 cm electrodes until they reached the size 2 × 2 cm. This was checked with the producer of the electrode to ensure that the electrodes worked in the same way after trimming/cutting them.

**Fig. 1:**
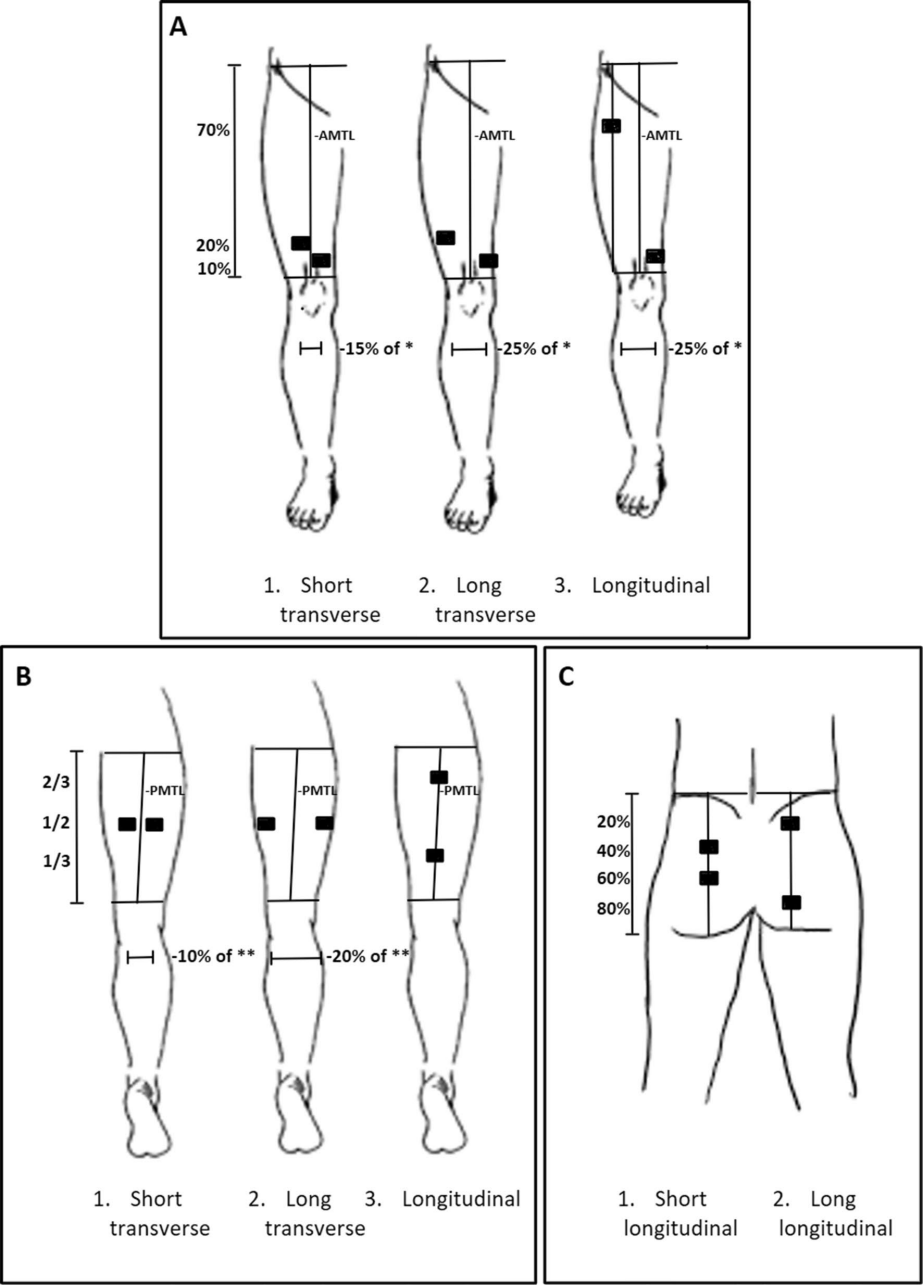
Placement of the electrodes on **A** quadriceps, **B** hamstrings and **C** gluteus. AMTL, anterior mid-thigh line; PMTL, posterior mid-thigh line. *Circumference of the thigh at 15% of the AMTL. **Circumference of the thigh at 50% of the PMTL

The 5 × 9 cm electrodes, had two possible attachments for each electrode so two cables could be attached to those electrodes, therefore a test with both one and two cables were performed on these electrodes at each position. However, the comfort according to NRS (0–10) was similar for the tests with one and two cables on each muscle group (quadriceps [*p* = 0.35], hamstrings [*p* = 0.73], gluteus maximus [*p* = 1.0]). Since only one cable could be attached to the smaller sized electrodes, only the data from the tests with one cable for the 5 × 9 cm electrodes were used and presented in this study. Since the 5 × 9 cm electrodes could be placed in either a transverse or longitudinal orientation [11] a test for each orientation was performed for these electrodes at each position, e.g. the electrodes were placed in a longitudinal orientation as well at each of the transverse orientations displayed in Fig. 1.

### Electrode placement

The placements of electrodes on the quadriceps [2, 11, 12, 20–24] and hamstrings muscles [20, 21] were based on previous studies on electrode placements as well as the recommended placements of the NMES-device. The placements used on the gluteus muscle were solely based on the recommendations of the NMES-device since no previous studies investigating electrode placements on the gluteus were found. Specific percentages based on the length and circumference of the thigh and gluteals of each participant were used in order to obtain as anatomically equal electrode placements as possible. The specific percentages used for each muscle group were based on our own pilot experiments, aiming to optimize the results of stimulation as described below.

#### Placement of the electrodes on the quadriceps (Fig. 1A)

The electrodes on the quadriceps were placed over the vastus medialis and lateralis. In order to determine the placement of the electrodes on each participant, anatomical landmarks were marked out at the upper border of the patella and at the point where the line between the left and right spina iliaca anterior superior intersected the longitudinal line running from the upper border of the patella. The straight line connecting these two anatomical landmarks was defined as the “anterior mid-thigh line” (AMTL). Then, the circumference of the thigh was measured at 15% of the length of the AMTL in proximal direction from the patella. The following electrode placements in medial and lateral direction from the AMTL were based on this circumference regardless of where on the thigh the electrodes were placed.

Three levels of electrode placements along the AMTL were marked, at 10%, 20% and 70% of the length of the AMTL in proximal direction from the patella, corresponding to the distal vastus medialis as well as distal and proximal vastus lateralis. For the short transverse placement (ST) and the long transverse placement (LT), a distance from AMTL of 15% and 25% of the circumference respectively, were marked out at 10% of the length of the thigh medially and 20% of the length of the thigh laterally for the placement of electrodes. For the longitudinal placement (L), the placement of the proximal electrode was marked at 70% of the length of the thigh in proximal direction from the patella, and at 25% of the circumference of the thigh in lateral direction from the AMTL. The distal electrode for the L-placement was placed at the same location as the medial electrode of the LT.

#### Placement of the electrodes on the hamstrings (Fig. 1B)

The electrodes over the hamstrings were placed to activate biceps femoris and semitendinosus. To determine the placement of the electrodes on each participant, anatomical landmarks were marked out at the middle of the popliteal fossa and the gluteal sulcus. The straight line, parallel to the longitudinal midline of the participant´s body, connecting these two anatomical landmarks was defined as the “posterior mid-thigh line” (PMTL) and the length of the posterior thigh was defined as the length of PMTL.

Three levels of electrode placement along the PMTL were marked, at 33%, 50% and 66% of the length of the PMTL. For the L-placement, the placements of the electrodes were marked along the PMTL at 33% and 66% length of the thigh. For the ST-placement and LT-placement, the circumference of the thigh was measured at a level corresponding to 50%& of the length of the thigh. Then, with a distance of 10% (ST) and 20% (LT) of the circumference respectively, the electrode placements were marked out at 50% of the length of the posterior thigh.

#### Placement of the electrodes on the gluteus maximus (Fig. 1C)

In order to determine the placement of the electrodes on the gluteus maximus, anatomical landmarks were marked out at the middle of the transverse gluteal fold and also at the point where a line between the left and right iliac crest intersected the line running from the middle of the gluteal fold, parallel to the longitudinal midline of the participant´s body. The length of this line was measured, and electrode placements were marked along this line at 40% and 60% (short longitudinal placement (SL)) as well as 20% and 80% (long longitudinal placement (LL)).

### Test procedure

The same investigator performed all the tests, on all participants. All tests were performed on either the left or the right leg, which was randomized for each participant. However, if the participant had an injury or had previous surgery on one leg, the other leg was used for the tests. Ten participants were tested on the right leg and five on the left leg. There were no significant correlations between the side of the leg tested and any of the outcome variables. The skin was not shaved prior to the testing and the participants were asked to not shave their thighs/gluteals two days prior to the testing. Prior to testing, the skin on the leg used for the test was washed with water and thoroughly dried with a towel. The different placements of the electrodes were marked with the participants standing up. Thereafter, the participant was seated down on a gurney. For the tests on the quadriceps, the participant was sitting with the knee flexed in approximately 30 degrees with a pillow placed under the knee. For the test on the hamstrings and gluteus maximus, the participant was lying down in prone position with the knee in full extension and hip in neutral position. The participants were instructed to relax fully during the stimulation. Each test started at the lowest NMES-level (0–999) of the device and was then gradually increased by two NMES-levels at a time. The NRS (0–10) was registered for each step and the NMES-levels where the first visible muscle twitch and the first visible muscle contraction occurred was registered. The first visible muscle twitch was defined as the first visible movement in any part of the muscle stimulated. The first visible muscle contraction was defined as the first visible coherent muscle contraction of the whole muscle. The test ended for each setting when a visible muscle contraction was reached. A short break (30 s) was provided between each test. There was a total of 44 different tests, with 16 different combinations for each of the quadriceps and hamstrings, and 12 different combinations on the gluteus maximus. To minimize the influence of the order of the tests, the order of the different muscle groups, electrode sizes and electrode placements was randomized on each participant. The 2 × 2 cm and 5 × 5 cm electrodes were tested on three placements on the quadriceps and hamstrings [6] and two placements on the gluteus maximus [4]. The 5 × 9 cm electrodes were placed in a vertical position on the different placements on all three muscle groups and also in a horizontal position on the LT- and L-placement on the quadriceps and hamstrings [5] as well as both the SL- and LL-placement on the gluteus maximus [4] and were also tested with one and two cables on all different placements [4].

#### First visible muscle twitch

For all the tests both the first visible muscle twitch and the first visible muscle contraction were registered. Based on previous studies using LI-NMES, the level equal to the first visible muscle contraction was thought to be most clinically relevant [5, 7]. The results from the first visible muscle twitch were used as a proxy prior to the first muscle contraction, with the purpose to confirm the results from the first visible muscle contraction. Therefore, the main results presented in this study are the NRS and the intensity required for the first visible muscle contraction, while the results for the first visible muscle twitch are presented in the Additional file 1: Data. All results for the muscle contraction, for each muscle group, electrode placement and size, were confirmed by the data collected at the first visible muscle twitch (see Additional file 1: Data).

### Statistical analysis

The sample size was determined prior to the start of the experiment using a power calculation based our own pilot study and a previous study [12], with the significance level set at *p* < 0.05 and power at 90% regarding the primary outcome, comfort, measured with the NRS. Based on a difference of 2 steps on the NRS [2–4], with a standard deviation of 2, as found in our pilot study between the different electrode configurations, 11 participants were needed to obtain a significant difference. We set the final sample size to 15 participants.

The data were analyzed using SPSS version 25 (IBM Corp. Released 2016. IBM SPSS Statistics, Armonk, NY) in cooperation with a statistician. Due to the relatively low number of participants (n = 15), and the fact that the majority of the outcomes was non-normally distributed according to the Shapiro-Wilks test, descriptive statistics were summarized using median, inter-quartile range and frequency, and the non-parametric Wilcoxon signed rank test was used for the inferential statistics. The data included some outliers, which was handled both by using a rank-based statistics test and by adjusting the values of the outliers to the lowest/highest value within 1.5 times the interquartile range from the first respectively third quartile for the inferential statistics [25]. To compare the effect on outcomes for different electrode sizes and placements, the outcome variable means for each size (including all the placements) and each placement (including all the sizes) were calculated. Then, the Wilcoxon signed rank test was conducted to detect if there were any statistically significant differences between the outcome means of the different electrode placements and sizes. The significance level in all analyses was set at *p* < 0.05.

## Results

### Electrode placement

#### Quadriceps

**Fig. 2:**
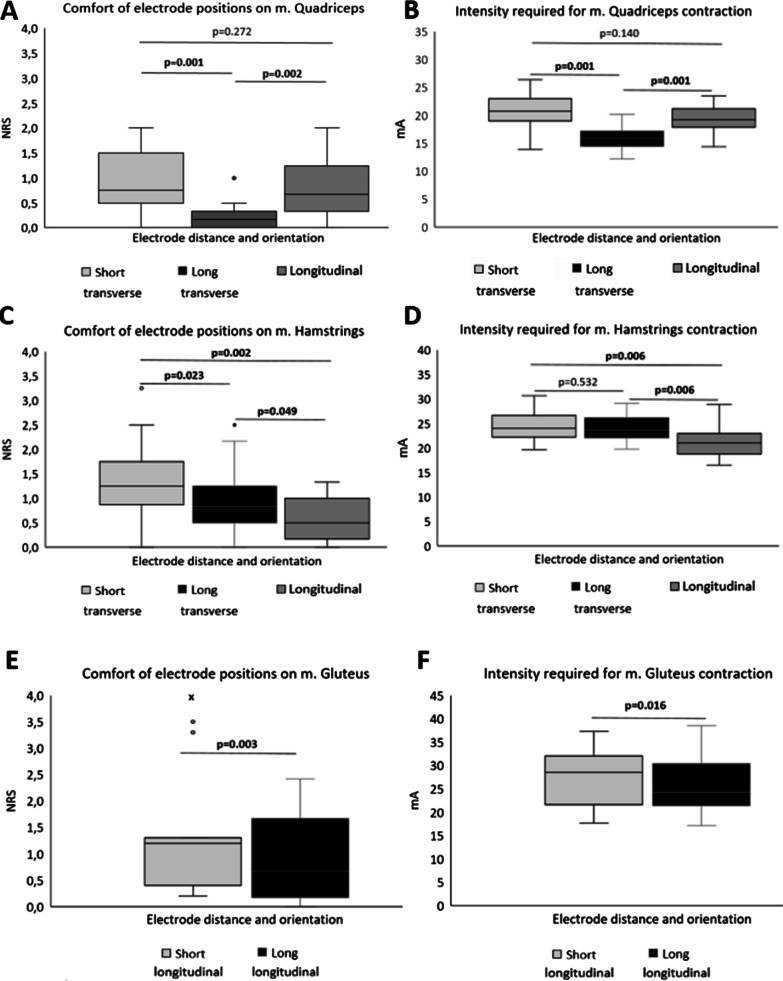
Comfort according to the Numeric Rating Scale for pain (NRS) and intensity needed for muscle contraction for different placements of the electrodes on quadriceps (**A**, **B**), hamstrings (**C**, **D**) and glutues (**E**, **F**). *P* values calculated with Wilcoxon signed rank test, significant values are bold

The most comfortable placement of the electrodes during LI-NMES induced muscle contraction on the quadriceps, according to the NRS (0–10), was the LT-placement which exhibited significantly better comfort compared to ST- and L-placements (median of 0.17 compared to 0.75 and 0.67, respectively) (Fig. 2A). In addition, among the placements, the LT required the lowest intensity for a muscle contraction (Fig. 2B).

#### Hamstrings

The L-placement resulted in significantly better comfort than the ST- and LT-placement according to NRS, median of 0.5 compared to 1.25 and 0.83, respectively, as shown in Fig. 2C. The L-placement also required significantly less intensity to produce a muscle contraction compared to the other placements (Fig. 2D).

#### Gluteus maximus

The LL-placement was significantly more comfortable compared to SL-placement according to NRS (median of 0.67 vs 1.2) (Fig. 2E) and the intensity required for a muscle contraction was also significantly lower (Fig. 2F). There were larger variations both in intensity and NRS on gluteus maximus compared to the other muscles investigated.

### Electrode size

#### Quadriceps

**Fig. 3:**
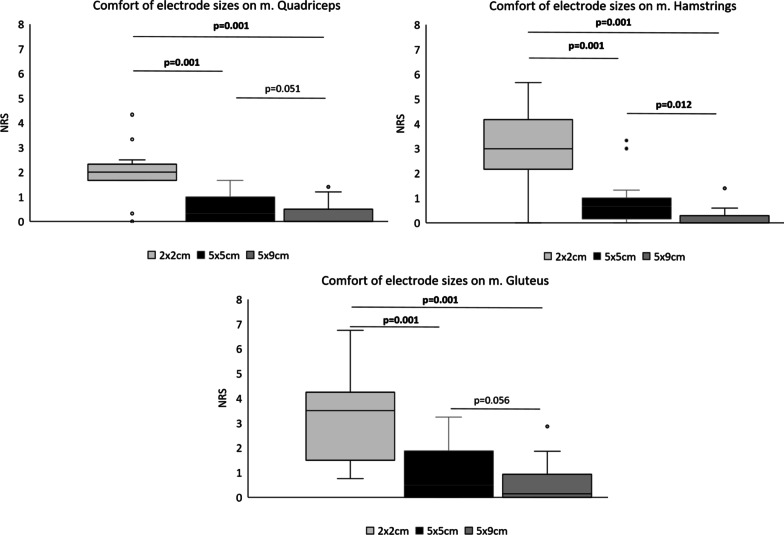
Comfort according to the Numeric Rating Scale for pain (NRS) of different electrode sizes (2 × 2 cm, 5 × 5 cm and 5 × 9 cm) on quadriceps, hamstrings and gluteus. P-values calculated with Wilcoxon sign rank test, significant values are bold

**Fig. 4:**
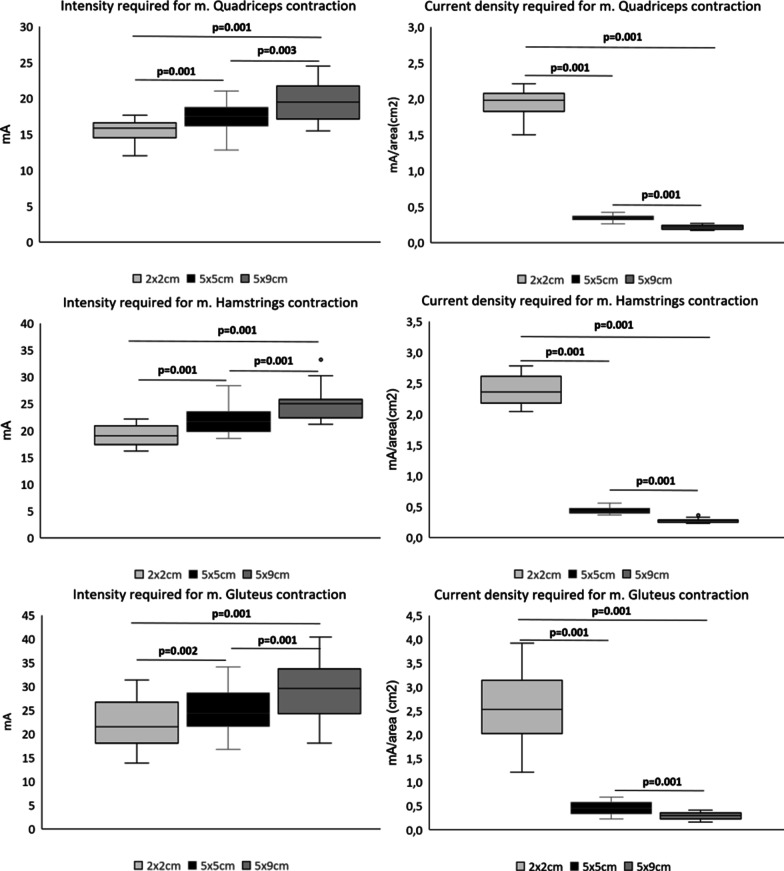
Intensity of NMES required for muscle contraction for the different electrode sizes (2 × 2 cm, 5 × 5 cm and 5 × 9 cm) on the quadriceps, hamstrings and gluteus. Intensity describes the total intensity (mA) and current–density describes the intensity/area (mA/cm^2^) needed for muscle contraction for each electrode size. *P* values calculated with Willcoxin sign rank test, significant values are bold. x = outlier (5.4) outside of the diagram

During LI-NMES on the quadriceps the 2 × 2 cm electrode was significantly less comfortable than the 5 × 5 cm and 5 × 9 cm electrodes, median NRS of 2 compared to 0.33 and 0, respectively. The 5 × 9 cm compared to the 5 × 5 cm electrodes exhibited better comfort, but the difference was not significant (*p* = 0.051) (Fig. 3). The intensity required for muscle contraction was significantly lower while the current–density was significantly higher for the 5 × 5 cm electrode compared to the 5 × 9 cm electrode (Fig. 4).

#### Hamstrings

The 5 × 9 cm electrode was significantly more comfortable than both the 2 × 2 cm and 5 × 5 cm electrodes, median NRS of 0 compared to 3 and 0.67, respectively (Fig. 3). The intensity required for muscle contraction was lower while the current–density was higher for the 5 × 5 cm electrode compared to the 5 × 9 cm electrode (Fig. 4).

#### Gluteus maximus

The 2 × 2 cm electrode was significantly less comfortable than both the 5 × 5 cm and 5 × 9 cm electrodes, median NRS of 3.5 compared to 0.5 and 0.14 respectively. The 5 × 9 cm compared to the 5 × 5 cm electrodes exhibited better comfort, but the difference did not reach significance (*p* = 0.056) (Fig. 3). The intensity required for muscle contraction was lower while the current density was higher for the 5 × 5 cm electrode compared to the 5 × 9 cm electrode (Fig. 4). There were larger variations in intensity required for muscle contraction on gluteus maximus compared to the other muscles investigated.

### Electrode orientation

The 5 × 9 cm electrodes were placed with two different orientations on all the positions, transverse and longitudinal orientation. There were, however, no significant differences of NRS or intensity needed for muscle twitch or muscle contraction either on the quadriceps, hamstrings or gluteus maximus for the two placements (Table 2).

**Table 2:** Comfort and intensity needed for muscle contraction for horizontal versus vertical placement of the 5 × 9 cm electrodes

	Quadriceps		Hamstrings		Gluteus	
	*p* value^a^		*p* value^a^		*p* value^a^	
NRS—vertical placement	0 (0–0.50)	0.91	0 (0–0.33)	0.53	0 (0–1.3)	0.58
NRS—horizontal placement	0 (0–0.38)		0.25 (0–0.50)		0 (0–0.44)	
Amplitude (mA)—vertical placement	20 (17–22)	0.39	24 (22–26)	0.57	29 (24–32)	0.21
Amplitude (mA)—horizontal placement	20 (17–22)		25 (22–26)		29 (23–37)	

NRS, Numeric Rating Scale for pain; mA, milli ampere

^a^*P* values calculated with Wilcoxon sign rank test. Data are expressed as median (inter-quartile range)

## Discussion

In this study we investigated different sizes and pragmatic placements of electrodes on three different muscle groups during LI-NMES, regarding comfort and intensity needed for muscle contraction. We found that the best sizes and placements of electrodes, out of the pre-determined placements tested, during LI-NMES differed between the quadriceps, hamstrings and gluteus maximus muscles, both regarding comfort and intensity required for muscle contraction. The superior electrode size and placement for LI-NMES were, the LT-placement with 5 × 5 cm electrodes on the quadriceps, the L-placement with 5 × 9 cm electrodes on the hamstrings and the LL-placement with 5 × 9 cm electrodes on the gluteus maximus.

The main results of this investigation demonstrated how the electrode placement influenced both the comfort and intensity required for muscle contraction during LI-NMES. The finding that a longer distance between the electrodes was, for each of the muscle groups tested, more comfortable and required lower intensity to induce a muscle contraction, is substantiated by other studies using HI-NMES [1, 13]. A previous study found, when comparing different distances between longitudinally placed electrodes on the quadriceps muscle at high intensities, that a longer distance produced a more pronounced knee extension [13]. Plausible physiological explanations for this finding may include that the electric current, with a longer distance between the electrodes, does not take the pathway through the subcutaneous layers but travels deeper in the muscle and moreover can recruit more muscle fibers. Partly in contrast to this, another study found that a relatively shorter distance between longitudinal placed electrodes produced a larger knee extension force (15 cm compared to 20–25 cm) [16]. However, the study did not investigate a shorter distance than 15 cm, as was done in this study with the ST-placement, and only used longitudinal placements of the electrodes. The observation on gluteus maximus that the LL-placement obtained better comfort and required less NMES current intensity than the SL-placement confirms that a longer distance between electrodes results in better outcome also on this muscle group, which has not been studied in this context before.

Our finding that the L-placement on the hamstrings was better than a transverse placement (LT and ST) is corroborated by other studies using HI-NMES [20, 21]. However, on the quadriceps muscle the LT-placement was more comfortable and required lower intensity for muscle contraction than the L-placement which contrasts with previous studies investigating HI-NMES. One reason to the discrepancy of the results is that previous studies used HI-NMES with stimulation intensities up to maximal level of tolerance [2, 10–13, 20, 21], whereas this study used LI-NMES until the first muscle contraction. A plausible explanation for that the LT-placement on the quadriceps was better than the other placements is that it is more likely to cover motor points. Thus, previous studies have indicated that motor points are more frequent in the distal-compared to the proximal quadriceps [14, 15].

An important aspect to consider is that this study analyzed various pre-determined commonly chosen placements of electrodes. Previous studies have shown that identification and placement of electrodes on motor points can minimize discomfort and intensity required for muscle contraction [2, 14, 15]. However, for patients using NMES at home, a motor point search can be quite difficult to perform, and rather pre-determined placements are more pragmatic to use. We therefore aimed to provide clear recommendations of where to place the electrodes when it is not possible to perform a motor point search. In addition, the observation that the best placements, on each muscle group, in this study resulted in NRS close to zero, suggests that these placements may be just as good as using motor points for the use of LI-NMES.

The second main finding of this study demonstrated that a smaller electrode size (2 × 2 cm) resulted in significantly less comfort than the larger electrode-sizes (5 × 5 cm and 5 × 9 cm), tested on each of the pre-determined placements on all three muscle groups, during LI-NMES. This finding is in line with several previous studies investigating HI-NMES [2, 9, 10, 20, 22]. Possible explanations are that larger electrodes are more likely to include motor points and that larger electrodes reduce the current density.

The observation on hamstrings that the 5 × 9 cm electrode was the most comfortable and required the lowest current density suggests in line with previous studies with high-intensity NMES that 5 × 9 cm is a good size for hamstring stimulation [9, 20]. On the quadriceps- and gluteus muscles however, the 5 × 5 cm and 5 × 9 cm electrode exhibited similar comfort, and the 5 × 5 cm electrode required lower total intensity but higher current density for muscle contraction than the 5 × 9 cm electrode. A possible explanation to the finding that the 5 × 9 cm compared to the 5 × 5 cm electrode only exhibited significantly better comfort on the hamstrings could be that the skin innervation on the posterior compared to anterior thigh and gluteals is more sensitive to NMES. On the quadriceps, the 5 × 9 cm electrode however unintentionally activated other muscles than the vastus lateralis and medialis, such as the hamstrings, which reduced the effectiveness of the stimulation which is in line with findings of previous studies [2, 12, 22]. Therefore, the 5 × 5 cm electrode was considered superior on the quadriceps. No previous studies have, to the best of our knowledge, compared different electrode sizes with LI-NMES or studied stimulation of the gluteus muscles. Our finding that 5 × 9 cm is a superior electrode size for the gluteus maximus is novel but is in line with general recommendations that larger muscles require larger electrodes. One study demonstrated findings contrary to ours and also to other previous studies, indicating that the size of the electrodes did not affect the comfort or effectiveness [12]. However, the small differences in sizes of electrodes used in that study (3.7 cm^2^, 4.4 cm^2^ and 5.1 cm^2^) as compared to this study (4 cm^2^, 25 cm^2^ and 45 cm^2^), may explain the discrepancies observed between the studies.

The comfort of the different electrode sizes in this study, according to the median NRS, varied between NRS 0-3.5, which is a large variation within clinically acceptable levels of discomfort. However, some individuals estimated the discomfort as high as NRS 8 on individual tests (if combining the SL-placement and 2 × 2 cm sized electrodes), and for example three participants reported a mean NRS over 6 for the 2 × 2 cm electrodes (including all placements) on the gluteus. The observation that the comfort for the different sizes and placements varied more at the level equal to muscle contraction compared to the level at the first visible muscle twitch, suggests that the variation in comfort of the different electrode sizes and placements may be even larger with HI-NMES.

One possible limitation of this study is that the muscle contractions were assessed by visible means, which means that the measurement is subjective and there are no previous studies that have performed intra- and inter-observer variation studies of these measurements. However, the same investigator performed all tests on all participants to control for observer bias. In addition, the differences in electrode placement and size observed at the muscle contraction were confirmed by similar results observed at the first visible muscle twitch (see Additional file 1: Results), which increased the reliability of the results. Previous studies of HI-NMES assessed knee extension torque with e.g. an isokinetic dynamometer or a force transducer measuring the movement in the limb [23, 24]. However, the LI-NMES used in this study was not high enough to be able to produce torque measurements. Another possible limitation is the fact that a total of 44 tests were performed during the same session with possibility of fatigue among the participants. However, to control for this the order of the test was randomized on each participant. One strength of this study is that it is the first study to investigate a total of 44 different combinations by varying both the electrode sizes and placements on the quadriceps, hamstrings and gluteals with LI-NMES. In addition, we used exact pre-determined placements based on each participant’s individual leg size.

## Conclusion

For LI-NMES-treatment we found specific pre-determined electrode placements and sizes that can be used with good comfort on each individual muscle of quadriceps, hamstrings and gluteus muscle. The best electrode configuration regarding comfort and efficiency was, the LT-placement with 5 × 5 cm electrodes on the quadriceps, the L-placement with 5 × 9 cm electrodes on the hamstrings and the LL-placement with 5 × 9 cm electrodes on the gluteus maximus.

## Supplementary Information

**Additional file 1:** The results for the first visible muscle twitch, for each muscle group, electrode placement and size.

## Data Availability

The datasets used and/or analyzed during the current study are available from the corresponding author on reasonable request.

## Abbreviations

AMTL: Anterior mid-thigh line
HI: High-intensity
LT: Long transverse placement
LL: Long longitudinal placement
LI: Low-intensity
NMES: Neuromuscular electrical stimulation
NRS: Numeric Rating Scale for pain
PMTL: Posterior mid-thigh line
L: Longitudinal placement
ST: Short transverse placement
SL: Short longitudinal placement
