# Supplementary material for: Effects of electrode size and placement on comfort and efficiency during low-intensity neuromuscular electrical stimulation of quadriceps, hamstrings and gluteal muscles
Source: BMC Sports Sci Med Rehabil. 2022 Jan 16;14:11. doi: 10.1186/s13102-022-00403-7 (PMC8761348; doi:10.1186/s13102-022-00403-7)
Supplement: Supplementary file 1 — Additional file 1: The results for the first visible muscle twitch, for each muscle group, electrode placement and size. [file 13102_2022_403_MOESM1_ESM.docx]

**Supplementary results – first muscle twitch**


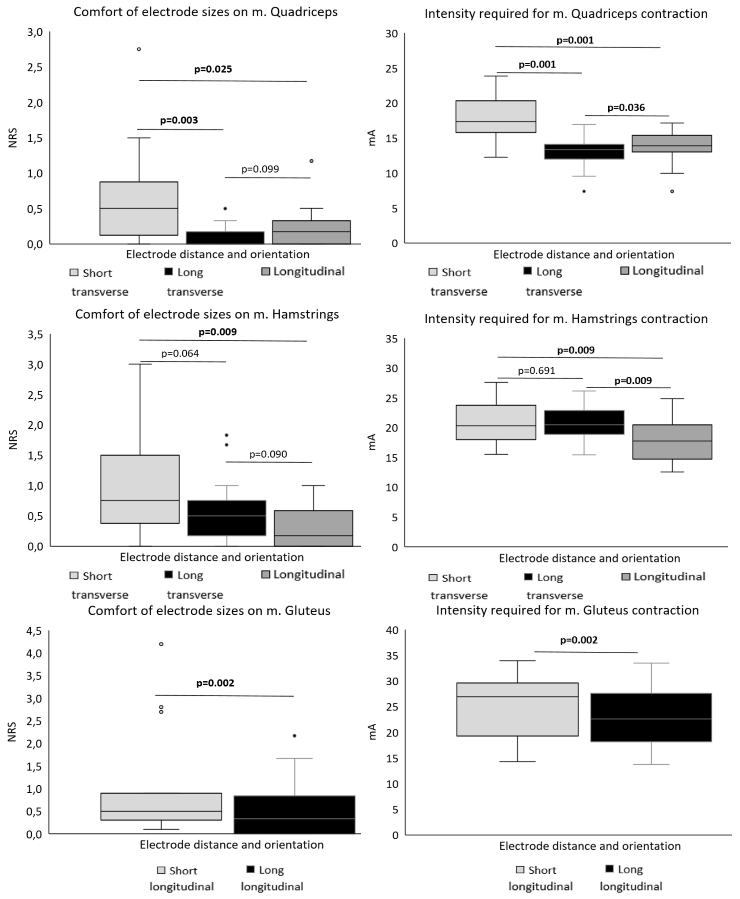
***Figure S1.*** ***Comfort according the Numeric Rating Scale for pain (NRS) and intensity needed for visible muscle twitch for different placements of the electrodes on the quadriceps, hamstrings and gluteus.*** *Intensity describes the total intensity (mA) needed for the first visible muscle twitch of the muscles for each electrode size. P-values calculated with Wilcoxon sign rank test, significant values are bold.*


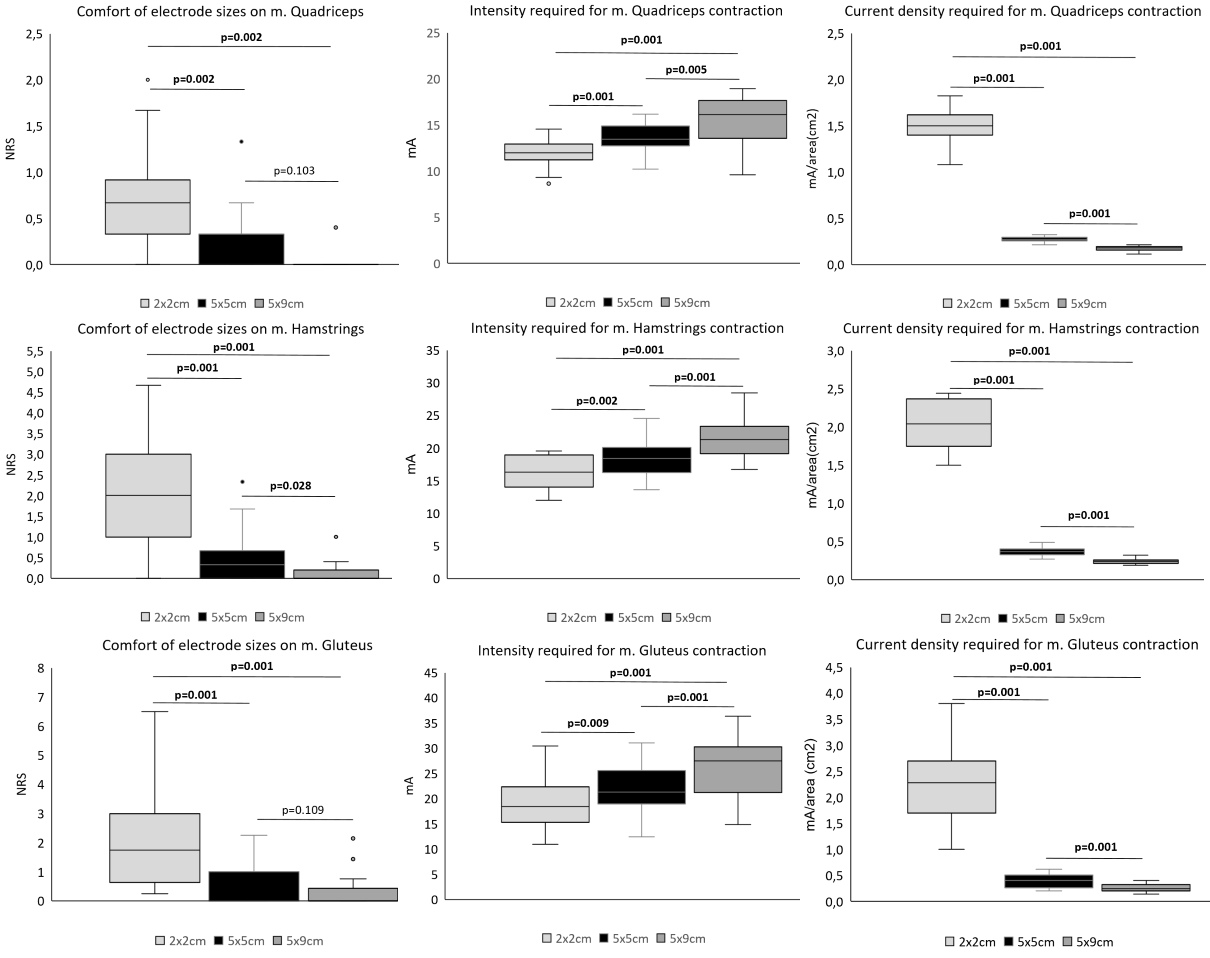


***Figure S2. Comfort according the Numeric Rating Scale for pain (NRS) and intensity of the NMES-stimulation require for first visible muscle twitch for the different electrode sizes (2x2cm, 5x5cm and 5x9cm) on the quadriceps, hamstrings and gluteus.*** *Intensity describes the total intensity (mA) and current density describes the intensity/area (mA/cm^2^) needed for the first visible muscle twitch of the muscles for each electrode size. P-values calculated with Wilcoxon sign rank test, significant values are bold.*

***Table S1. Comfort and intensity needed for first muscle for horizontal vs vertical placement of the 5x9cm electrodes.***

|  | Quadriceps | | Hamstrings | | Gluteus | |  |
| --- | --- | --- | --- | --- | --- | --- | --- |
|  |  | **p-value ^a^** |  | **p-value ^a^** |  | p-value ^a^ |  |
| NRS – vertical placement | 0 (0-0.09) | 0.068 | 0 (0-0) | 0.83 | 0 (0-0.75) | 0.60 |  |
| NRS – horizontal placement | 0 (0-0) |  | 0 (0-0.13) |  | 0 (0-0) |  |  |
| Amplitude (mA) – vertical placement | 16 (14-17) | 0.55 | 20 (19-22) | 0.41 | 27 (21-30) | 0.73 | |
| Amplitude (mA) – horizontal placement | 15 (13-17) |  | 21 (19-23) |  | 26 (20-31) |  |  |

**^a^** P-values calculated with Wilcoxon sign rank test. Data are expressed as median (inter-quartile range). NRS=Numeric Rating Scale for pain. mA=MilliAmpere.
